# Association of Psychological Characteristics and Functional Dyspepsia Treatment Outcome: A Case-Control Study

**DOI:** 10.1155/2016/5984273

**Published:** 2016-07-28

**Authors:** Yiping Chen, Caihua Wang, Jinyu Wang, Leilei Zheng, Weibo Liu, Huichun Li, Shaohua Yu, Bin Pan, Hualiang Yu, Risheng Yu

**Affiliations:** ^1^Department of Psychiatry, Zhejiang University School of Medicine Second Affiliated Hospital, Hangzhou, Zhejiang 310009, China; ^2^Department of Gastroenterology, Zhejiang University School of Medicine Second Affiliated Hospital, Hangzhou, Zhejiang 310009, China; ^3^Department of Epidemiology, Shandong University School of Public Health, Jinan, Shandong 250012, China; ^4^Department of Radiology, Zhejiang University School of Medicine Second Affiliated Hospital, Hangzhou, Zhejiang 310009, China

## Abstract

This study was to investigate the association of psychological characteristics and functional dyspepsia treatment outcome. 109 patients who met the criteria for FD were enrolled. Eysenck Personality Questionnaire (EPQ), Symptom Checklist 90 (SCL90), and the Pittsburgh Sleep Quality Index (PSQI) were used to measure personality, psychological symptoms, and sleep quality in our patients. Leeds Dyspepsia Questionnaire (LDQ) was used to assess dyspeptic symptoms at baseline and after eight weeks of treatment. The LDQ scores change after therapy, and the degraded rate of LDQ was used to assess the prognosis of patients. Logistic regression model was used to assess the effect of the personality, psychological symptoms, and sleep quality on the prognosis of patients. Our result revealed that poor sleep quality (OR = 7.68, 95% CI 1.83–32.25) and bad marriage status (OR = 1.22, 95% CI 1.10–1.36) had the negative effect on the prognosis of FD, while extroversion in personality traits (OR = 0.86, 95% CI 0.76–0.96) had positive effect on the prognosis of FD. We should pay attention to the sleep quality, the personality, and the marriage status of FD patients; psychological intervention may have benefit in refractory FD.

## 1. Introduction

Functional dyspepsia (FD) is a clinical disorder present with persistent or recurrent abdominal discomfort or pain located in the upper abdomen with unknown responsible structural lesions [1]. Recent studies indicated a heterogeneity of this disorder whose specific clinical symptom patterns could be related to varied gastric pathophysiological mechanisms [2]. Gastric motility determined by delayed gastric emptying or impaired accommodation and visceral hypersensitivity determined by abnormal sensitivity to gastric balloon distension have been suggested to be responsible for the dyspeptic symptoms involved in FD. Psychological factors have also been associated with FD. Clinical observations suggested a higher anxiety level and stress experienced in FD patients with a positive correlation to the disease severity [3]. Up to 87% of patients with FD had a psychiatric diagnosis in a clinic study [4]. Personality trait estimation revealed higher neuroticism score by Eysenck Personality Questionnaire [5]. Clinical data even pointed out a disordered sleep reported from patients with FD [6].

Previous literature posed several possible explanations for the association between psychological state and FD which could be grouped into two major categories: psychological state leads to FD; FD influences psychological state. For the former, it was thought that the psychological factors induced the onset of FD or FD was just the clinical presentation of the abnormal psychological difficulties [7]. The latter, however, centered on the influence of FD on psychological state or these psychological difficulties were secondary to the chronic symptoms of FD. Lately, some investigators tried to use the “Brain-Gut Axis” (BGA) to explain the link between psychological state and FD. This idea is a rough neurobiological framework for reciprocal connections between the brain and the gastrointestinal tract [8]. Current explanations are still at hypothesis stage and need more research for confirmation.

Though many clinical observations of psychological factors and FD have been carried out worldwide, few have been done in China. It is said that culture in China is different from other countries and the evidences from western countries may be not appropriate in China. In this study, we investigated Chinese patients diagnosed with FD in our center. We characterized their personality traits and sleep quality and depression and anxiety levels. Further analysis grouped these patients into two groups based on the response to anti-FD treatment and compared psychological traits between them. We assume that the severity of psychological problems (poor sleep quality, personality, and psychological symptoms) is correlated with the response to treatments of FD.

## 2. Materials and Methods

### 2.1. Patient Sample

109 patients diagnosed with FD during June 2014 and December 2014 were enrolled. All patients presented to the outpatient clinic of the Gastroenterology Department at the Second Affiliated Hospital of Zhejiang University School of Medicine. Detailed information of patients was got from clinical medical records and questionnaire. Inclusion criteria were as follows: (1) having fulfilled Rome III criteria for FD [9]; (2) the fact that presence of FD must be for at least 12 weeks during the last 6 months: severity of FD must be above moderate with the Leeds Dyspepsia Questionnaire (LDQ) [10] score more than 8; (3) the absence of organic, systemic, or metabolic disease; and (4) being negative in* Helicobacter pylori* infection. Exclusion criteria were as follows: (1) presence of esophagitis, gastric atrophy, erosive gastroduodenal lesions on endoscopy, heartburn as a predominant symptom, a history of peptic ulcer, and major abdominal surgery and (2) the use of nonsteroidal anti-inflammatory drugs, steroids, or drugs affecting gastric acid secretion.

All the subjects were acquainted in detail with the study procedure and they all signed a written consent form. The study was approved by the Ethic Committee of the Second Affiliated Hospital of Zhejiang University School of Medicine. All the subjects underwent psychological examinations which included psychological evaluation by a specialist of psychiatry. Treatment focused on the most bothersome symptoms for eight weeks, thus a prokinetic (i.e., domperidone) or antisecretory agent (i.e., esomeprazole) was prescribed in standard dose. Treatment could also include herbal preparation.

### 2.2. Questionnaires

Self-report questionnaires were used to collect the information of FD patients. Eysenck Personality Questionnaire (EPQ), Symptom Checklist 90 (SCL90), and the Pittsburgh Sleep Quality Index (PSQI) were used to measure personality, psychological symptoms, and sleep quality in our patients. We used the LDQ to assess FD symptoms at baseline and after eight weeks of treatment.

EPQ [11] included three dimensions: extroversion (*E*), neuroticism (*N*), and psychoticism (*P*). *E* scores range from 0 to 21, where the high score suggests the outgoing personality; *P* scores range from 0 to 20, where the high score indicates stubborn personality; *N* scores range from 0 to 24, where the high score suggests neurotic personality. EPQ has been widely used in China and has been proved validated [12].

SCL90 [13] includes nine factors, which are psychotic somatization (F1), obsessive-compulsive symptoms (F2), interpersonal sensitivity (F3), depression (F4), anxiety (F5), hostility (F6), terror (F7), phobic anxiety (F8), and psychoticism (F9). The item of the scale is assessed in reference to how much discomfort there is of the particular problem during the past 7 days. The scale accessed the psychological symptoms of the FD patients. SCL90 has good reliability and validity in Chinese population [14].

PSQI [15] is a self-rated questionnaire which assesses sleep quality and disturbances over a 1-month time interval; it is made up of seven aspects: subjective sleep quality, sleep latency, sleep duration, habitual sleep efficiency, sleep disturbances, use of sleeping medication, and daytime dysfunction. The sum of scores for these seven components yields one global score, the total scores range from 0 to 21, and the higher score stands for poorer sleeping quality. Chinese version of PSQI has been proved validated in previous study [16].

We used the LDQ to assess dyspeptic symptoms at baseline and after eight weeks of treatment. The scale measures eight dyspepsia symptoms with six grades each (where a grade of 0 indicates not present, 1 indicates very mild, 2 indicates mild, 3 indicates moderate, 4 indicates severe, and 5 indicates very severe); a summary score with a range of 0 to 40 represents the severity of dyspepsia. It contains epigastric pain, retrosternal pain, regurgitation, nausea, vomiting, belching, early satiety, and dysphagia; those that did not have dyspepsia according to questions 1–5 scored 0. LDQ scores 1–4 were classified as very mild dyspepsia, scores 5–8 were classified as mild dyspepsia, scores 9–15 were classified as moderate dyspepsia, and scores >15 were classified as severe or very severe dyspepsia. We then grouped our patients into two groups according to the LDQ scores change after therapy, and the degraded rate of LDQ ((the LDQ score before treatment − the LDQ score after treatment)/the LDQ score before treatment) was used to assess the prognosis of patients. The patients with degraded rate of LDQ more than 50% were classified into improvement group, while the individuals with degraded rate of LDQ less than 50% were classified into nonimprovement group. The Chinese version of LDQ is validated used in Chinese study [17].

### 2.3. Statistical Analysis

All data are described by means ± standard deviation (SD) and percentages. Two groups of numerical variables were compared by *t*-test, and classification variables were compared by *χ* ^2^-test. Multivariate nonconditional logistic regression model was used to screen the significant factors. The prognosis of patients (classified into improvement and nonimprovement) was set as dependent variable and demographic variables and psychological variables were set as independent variables. The multivariate model included variables that had statistical significance in univariate analysis, and stepwise method was used in regression. Statistical significance was considered with *P* < 0.05. All statistical analysis was conducted by SPSS version 18.0.

## 3. Results

### 3.1. Demographic Data

There are 109 patients (36 males, 73 females) in our study. The mean duration of FD symptoms was 112 ± 95 months, and the mean age is 47.56 ± 11.33 years. The good marriage in improvement group was higher than the nonimprovement group (92.86% versus 70.15%, *χ* ^2^ = 8.00,  and *P* = 0.005). There are no significant differences in other general demographic characteristics. Detailed information is shown in Table 1.

Data of SCL90 in patients with nonimprovement after treatment reported significantly higher score in dimensions F1, F2, F3, F4, F5, F7, F8, and F9 compared to those in effective group. The EPQ score in *E* dimension is significantly higher in patients with improvement, while *N* dimension is significantly higher in patients with nonimprovement. The PSQI score in nonimprovement group is significantly higher. Detailed comparison is shown in Table 2.

The result of multivariate logistic regression analysis revealed that the PSQI total score (OR = 1.22, 95% CI 1.10–1.36), another marriage status (OR = 2.04, 95% CI 1.83–32.25), and *E* dimensions of EPQ (OR = 0.86, 95% CI 0.76–0.96) had significant effect on LDQ outcome (Table 3).

## 4. Discussion

FD is a defined heterogeneous clinical entity characterized by recurrent or chronic dyspeptic symptoms in the gastroduodenal region, without organic lesions revealed on standard diagnostic modalities [1]. We found that prognosis might be influenced by the marriage status, personality characteristics, and sleep quality in Chinese FD patients.

FD patients have a variety of upper gastrointestinal symptoms including upper abdominal pain, reflux, vomiting, retrosternal burning, nausea belch, and early satiety. The pathophysiology for FD remains unclear, and several possible explanations have been presented in these years including visceral hypersensitivity and abnormal gastric motility [18]. Maybe a good marriage status provides the patients with a good social support and relieves the psychological pressure in patients. Psychological pressure, such as anxiety, has negative impact on FD patients [19].

Moreover, it has been reported that psychosocial factors may be more important than gastric sensorimotor dysfunction in the severity of dyspeptic symptoms [20]. However, the association between psychopathology and personality is heterogeneous among FD patients, reflecting the diverse underlying pathophysiological mechanisms or diversity in different populations.

The impact of sleep disorders on FD patients has received significant attention in recent years. Sleep disturbances may influence cognitive performance of FD patients including the problem solving and decision-making ability, moral reasoning, attention span, and reaction time [21, 22]. Whether this association is reflecting a comorbid condition, a predisposition, a care-seeking behavior, or a specific acting mechanism remains obscure, but the relationship of abnormalities in rapid eye movement (REM) sleep to autonomic nervous system function has been proposed [23, 24]. Obviously, the association between sleep disturbances and FD symptoms is complex. Intuitively, dyspeptic symptoms could interfere with sleep by preventing or delaying its onset and interrupting its continuation. But the reverse, disrupted sleep potentially also leads to increased symptom expression in patients with FD. Sleep deprivation has been shown to decrease thresholds of pain in healthy volunteers [25].

Personality traits (irritability, aggression, negativism, and neuroticism) have been early investigated in FD patients [26]. In our study, the introversion-extroversion is one of the most important factors of personality traits influencing the FD prognosis. For years, native Chinese people were labeled as “shy,” introverted, which is deeply influenced by our culture. We believed that these culture differences and deep personality characteristics would actually influence our understanding of the psychological facets of FD in a specific population.

According to our findings, in the treatment of FD patients, we should also pay attention to their sleep and improve sleep quality. Meanwhile, the personality and the marriage status of the patients should be taken into consideration; psychological intervention may have benefit in this specific population of FD patients.

There are some limitations in our study. First, the sample size is small. Some psychological symptoms may have effects on the prognosis of FD patients. Second, there may be some interactions among the sleep quality, psychological status, and personality traits; the impact of such interactions was not evaluated in the current study. We need to include a larger number of subjects in future FD clinical trials to evaluate the relationships between the different prognosis and psychological characteristics and further evaluate the concept of whether psychological intervention could have benefit in FD patients not responding to conventional therapy.

## 5. Conclusions

In summary, this study demonstrates that sleep quality and marriage status and introversion-extroversion of personality traits significantly influence the conventional treatment effects on Chinese FD patients. Future trials need to further evaluate the concept of whether psychological intervention could have benefit in FD patients not responding to conventional therapy and determine the cost-effectiveness of this approach.

## Figures and Tables

**Table 1:** Demographic characteristics of Chinese FD patients.

	Improvement (*N* = 57) Mean ± SD/*n* (%)	Nonimprovement (*N* = 52) Mean ± SD/*n* (%)	*t*/*χ* ^2^	*P* value
Age (years)	47.14 ± 11.79	47.82 ± 11.11	−0.30	0.763
Gender			0.22	0.637
Male	15 (35.71)	21 (31.34)		
Female	27 (64.29)	46 (68.66)		
BMI			2.07	0.355
<18.5	5 (11.90)	12 (17.91)		
18.5–25	35 (83.33)	48 (71.64)		
>25	2 (4.76)	7 (10.45)		
Marriage status			8.00	**0.005**
Good marriage	39 (92.86)	47 (70.15)		
Others	3 (7.14)	20 (29.85)		
Occupation			0.35	0.553
Employed	25 (59.52)	36 (53.73)		
Others	17 (40.48)	31 (46.27)		
Education level			0.02	0.883
Lower than high school	27 (64.29)	44 (65.67)		
High school and above	15 (35.71)	23 (34.33)		

**Table 2:** The comparison of psychological status in different prognosis groups patients.

Psychological status		Improvement (*n* = 57)	Nonimprovement (*n* = 52)	*t*	*P*
SCL90	F1	1.71 ± 0.55	2.13 ± 0.64	−3.47	**0.001**
	F2	1.56 ± 0.47	1.95 ± 0.67	−3.27	**0.001**
	F3	1.33 ± 0.49	1.70 ± 0.63	−3.27	**0.001**
	F4	1.50 ± 0.47	1.94 ± 0.68	−3.69	**<0.001**
	F5	1.54 ± 0.62	1.91 ± 0.57	−3.15	**0.002**
	F6	1.50 ± 0.57	1.69 ± 0.62	−1.56	0.122
	F7	1.21 ± 0.31	1.54 ± 0.58	−3.40	**0.001**
	F8	1.38 ± 0.52	1.62 ± 0.57	−2.18	**0.031**
	F9	1.38 ± 0.40	1.59 ± 0.53	−2.11	**0.037**

EPQ	*P*	5.19 ± 1.85	5.28 ± 2.18	−0.23	0.819
	*E*	10.55 ± 4.62	7.64 ± 3.91	3.52	**0.001**
	*N*	10.33 ± 5.50	13.37 ± 5.70	−2.75	**0.007**

PSQI total score		6.69 ± 3.89	11.09 ± 5.24	−4.69	**<0.001**

*Notes*. SCL90: symptom checklist 90; F1: psychotic somatization; F2: obsessive-compulsive symptoms; F3: interpersonal sensitivity; F4: depression; F5: anxiety; F6: hostility; F7: terror; F8: phobic anxiety; F9: psychoticism; EPQ: Eysenck Personality Questionnaire; *E*: extroversion, *N*: neuroticism; *P*: psychoticism; and PSQI: Pittsburgh Sleep Quality Index.

**Table 3:** The multivariate logistic regression model of the influence factors of FD prognosis.

	B	SE	OR	95% CI	*P*
Marriage (others versus good marriage)	2.04	0.73	7.68	1.83–32.25	**0.005**
PSQI total score	0.20	0.06	1.22	1.10–1.36	**<0.001**
EPQ *E*	−0.15	0.06	0.86	0.76–0.96	**0.008**

## Abbreviations

FD: Functional dyspepsia
EPQ: Eysenck Personality Questionnaire
SCL90: Symptom Checklist 90
PSQI: Pittsburgh Sleep Quality Index
LDQ: Leeds Dyspepsia Questionnaire
BGA: Brain-Gut Axis
REM: Rapid eye movement
SD: Standard deviation.
